# Regional differences in APD restitution can initiate wavebreak and re-entry in cardiac tissue: A computational study

**DOI:** 10.1186/1475-925X-4-54

**Published:** 2005-09-20

**Authors:** Richard H Clayton, Peter Taggart

**Affiliations:** 1 Department of Computer Science, University of Sheffield, Regent Court, 211 Portobello Street, Sheffield S1 4DP, UK; 2 Departments of Cardiology and Cardiothoracic Surgery, University College Hospital, 16-18 Westmoreland Street, London W1G 8PH, UK

## Abstract

**Background:**

Regional differences in action potential duration (APD) restitution in the heart favour arrhythmias, but the mechanism is not well understood.

**Methods:**

We simulated a 150 × 150 mm 2D sheet of cardiac ventricular tissue using a simplified computational model. We investigated wavebreak and re-entry initiated by an S1S2S3 stimulus protocol in tissue sheets with two regions, each with different APD restitution. The two regions had a different APD at short diastolic interval (DI), but similar APD at long DI. Simulations were performed twice; once with both regions having steep (slope > 1), and once with both regions having flat (slope < 1) APD restitution.

**Results:**

Wavebreak and re-entry were readily initiated using the S1S2S3 protocol in tissue sheets with two regions having different APD restitution properties. Initiation occurred irrespective of whether the APD restitution slopes were steep or flat. With steep APD restitution, the range of S2S3 intervals resulting in wavebreak increased from 1 ms with S1S2 of 250 ms, to 75 ms (S1S2 180 ms). With flat APD restitution, the range of S2S3 intervals resulting in wavebreak increased from 1 ms (S1S2 250 ms), to 21 ms (S1S2 340 ms) and then 11 ms (S1S2 400 ms).

**Conclusion:**

Regional differences in APD restitution are an arrhythmogenic substrate that can be concealed at normal heart rates. A premature stimulus produces regional differences in repolarisation, and a further premature stimulus can then result in wavebreak and initiate re-entry. This mechanism for initiating re-entry is independent of the steepness of the APD restitution curve.

## Background

Understanding the mechanisms that initiate and sustain malignant ventricular arrhythmias is an important research problem because ventricular tachycardia and fibrillation (VT and VF) are a notable cause of premature death, and remain an important public health problem in the industrialised world. Recent attention has focussed on restitution, the influence of an abrupt change in cycle length on action potential duration (APD). The dynamic behaviour of APD in response to cycle length changes has been shown in theoretical, experimental and computational studies to be a major determinant of wavefront stability [1,2]. An APD restitution curve with a slope > 1 can result in the initiation and subsequent instability of re-entrant arrhythmias [3], although other important mechanisms of initiation and instability have also been identified [4].

Regional differences in electrophysiological properties are a characteristic finding in the hearts of patients with cardiac pathology. Regional differences in repolarisation are often described as dispersion, and vulnerability to arrhythmias has been shown to depend on dispersion of repolarisation in both experimental [5,6] and computational studies [7-10]. This conceptual link between dispersion of repolarisation and vulnerability to re-entry may make a tacit assumption that APD dispersion is a static property of the tissue, resulting from underlying heterogeneity in electrophysiology. However, APD is a dynamic property of cardiac tissue, and is reduced at short diastolic interval (DI). The APD restitution curve is a model of the dynamical behaviour of cardiac tissue. One important consequence of this dynamic behaviour is that dispersion of repolarisation can be produced in electrophysiologically homogenous tissue if the DI is spatially non-uniform [11,12], resulting in alternans, wavebreak and re-entry.

Regional differences in both static and dynamic APD are a property of normal cardiac tissue. Regional differences in APD restitution have been documented, both at different locations within the ventricular wall [13-15], between left and right ventricle [16], and in chronically ischaemic hearts [17]. Experimental optical mapping of the ventricular epicardial surface has shown that regional differences in APD restitution can be exposed by a closely coupled premature stimulus [18], and that dispersion of repolarisation produced in this way increases vulnerability to VF [16,19,20].

**Figure 1:**
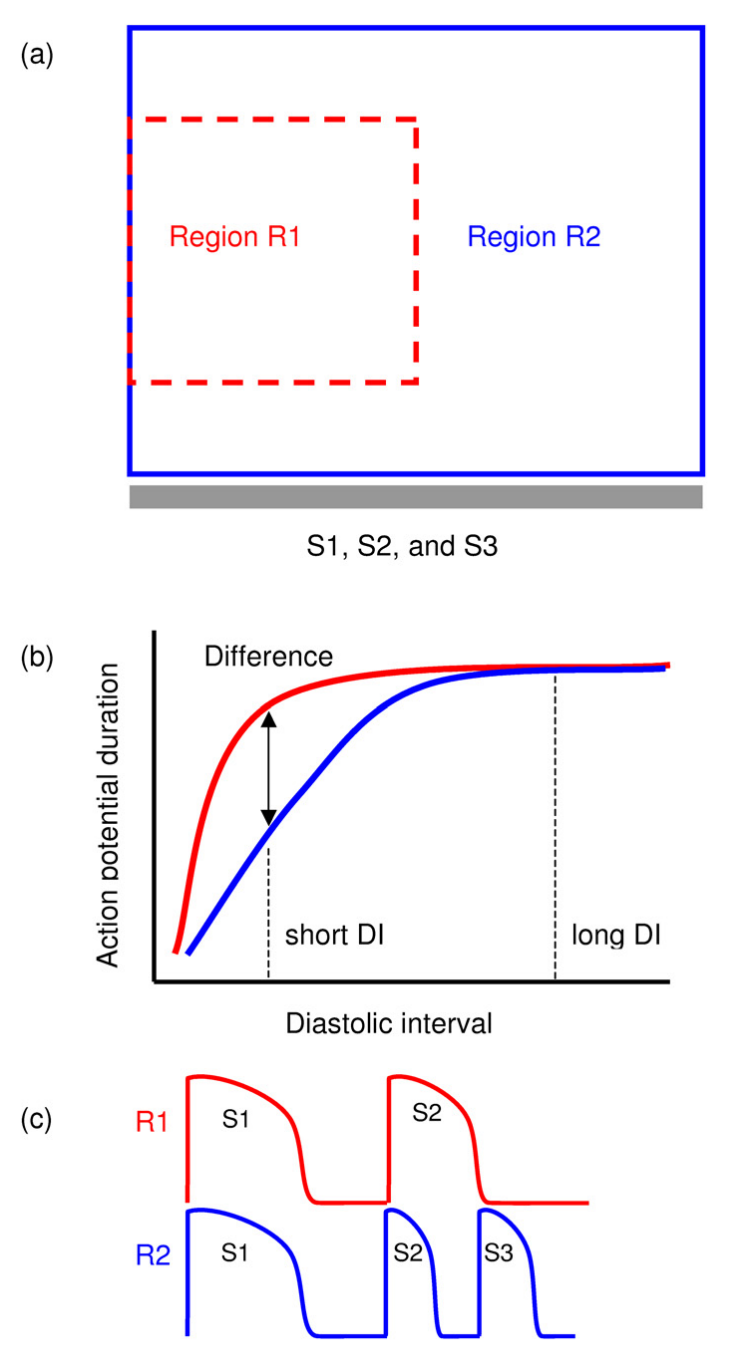
Idealised arrangement of cardiac tissue with regional differences in APD restitution. (a) Two-dimensional tissue sheet, with regions R1 and R2 outlined in red and blue respectively, and stimulus electrode at the bottom. (b) Cartoon of APD restitution of regions R1 (red) and R2 (blue), showing APDs in each region resulting from stimulus at long and short DI. (c) Cartoon of action potentials elicited in R1 and R2 with S1 S2 S3 stimulus protocol.

Figure 1(a) illustrates a simplified caricature of cardiac tissue with two neighbouring regions, each with the APD restitution curve shown in Figure 1(b). In the first region (R1) the APD restitution curve is flat across a wide range of DI with a steep decline at short DI, whereas in the second region (R2) the APD restitution has a shallower slope. If we assume that the conduction velocity (CV) restitution of regions R1 and R2 is the same, then pacing the tissue at a long cycle length, and hence with a long DI, will elicit a similar APD in each region as indicated in Figure 1(b). A premature stimulus (S2) with a short DI will, however, elicit a different APD in R1 and R2, as indicated in Figure 1(b). An additional premature stimulus (S3) could then encounter a long APD in R1 and a short APD in R2. If the S2S3 interval is sufficiently short, then this stimulus could be blocked in R1, resulting in wavebreak at the R1R2 boundary. This is illustrated in Figure 1(c), which shows a cartoon of action potentials in R1 and R2. Wavebreak is a precursor to re-entry, and so a sequence of three stimuli (S1, S2, S3) delivered from the same site would initiate wavebreak and then re-entry in tissue with this type of regional difference in APD restitution.

The behaviour of cardiac tissue with regional differences in APD restitution has not been studied in detail. The novel aspect of this study was therefore to examine the idealised situation shown in Figure 1, and to investigate how wavebreak and re-entry can be produced in tissue with regional differences in APD restitution. A wider aim of this study was to assess whether vulnerability to arrhythmias could be predicted from knowledge of the APD restitution properties of the tissue in a particular heart. Computational models of cardiac tissue are becoming a valuable experimental tool for testing and proposing hypotheses because tight control over tissue geometry and electrophysiology enables some problems to be dissected into their component parts. We therefore used a simplified computational model of cardiac tissue in which the APD restitution of R1 and R2 could be varied in a controlled manner.

## Methods

### Computational model

We simulated action potential propagation in a 2D sheet of isotropic cardiac tissue with membrane voltage *V*_*m *_described by the monodomain equation



where *C*_*m *_is specific membrane capacitance, D a diffusion coefficient and *I*_*ion *_current flow though the cell membrane per unit area. A great many cell models have been developed that reproduce the action potential of cardiac cells from different species, and from different parts of the heart [21]. Biophysically detailed models are computationally demanding to solve, and so we elected to use a simplified model of the action potential. For this study the key feature of cardiac electrophysiology was the APD and CV restitution, and this is captured by the 3-variable model described by Fenton and Karma [4,22,23], which we used to describe *I*_*ion*_. Details of the model are given in Appendix 1. We used the four parameter sets given in Table 1[4,22] to give four variants of the model, each with different APD restitution. The first two variants (*Steep1 *and *Steep2*) had an APD restitution slope > 1 at short DI, and the second two (*Flat1 *and *Flat2*) had an APD restitution slope < 1 at short DI.

**Table 1:** Parameter values for each variant of the 3-variable model

Parameter	*Steep1*	*Steep2*	*Flat1*	*Flat2*	units
*V*_*0*_	-85	-85	-85	-85	mV
*V*_*fi*_	15	15	15	15	mV
*g*_*fi*_	4	4	4	4	mS cm^-2^
*t*_*d*_	0.25	0.25	0.25	0.25	ms
*t*_*r*_	50	33	33	33	ms
*t*_*si*_	45	30	30	30	ms
*t*_*0*_	8.3	12.5	12.5	12.5	ms
*t_v_*^+^	3.33	3.33	3.33	3.33	ms
*t_v1_*^-^	1000	1250	1250	1250	ms
*t_v2_*^-^	19.2	19.6	19.6	19.6	ms
*t_w_*^+^	667	870	870	870	ms
*t_w_*^-^	11	41	60	120	ms
*u*_*c*_	0.13	0.13	0.13	0.13	None
*u*_*v*_	0.055	0.04	0.04	0.04	None
*u_c_^si^*	0.85	0.85	0.85	0.85	None
*k*	10	10	10	10	None

### Numerical methods

We solved the FK equations using a simple explicit Euler scheme, and the nonlinear diffusion equation using a forward time centre space finite difference method with a time step (Δt) of 0.1 ms, a space step (Δ) of 0.25 mm and no-flux boundary conditions at each edge. The specific membrane capacitance was set to 1 μF cm^-2^, and the diffusion coefficient set to 0.1 mm^2 ^ms^-1^.

### Tissue geometry and stimulus protocol

We examined the initiation of re-entry in 150 × 150 mm 2D tissues with R1 and R2 allocated the steep APD restitution variants *Steep1 *and *Steep2 *respectively, and with square and circle configurations of R1. We then repeated the study with R1 and R2 allocated the flat restitution variants *Flat1 *and *Flat2 *respectively. For the square configuration, region R1 was defined for *x *<*nx/2 *and *ny*/*4 *<*y *<*3ny*/*4*, with region R2 elsewhere. This arrangement corresponded to the idealised geometry shown in Figure 1. In the circle configuration we sought to simulate a more physiologically plausible arrangement, and so we allocated R1 to a circular region located in the centre of the tissue, with a radius of 12.5, 25, or 50 mm.

Each tissue sheet was stimulated along its bottom edge (*y = 0*) by raising the bottom 0.5 mm of tissue above threshold for 2 ms with an S1 S2 S3 stimulation protocol. The initial conditions for the model were imposed as described above to correspond to the state of the model following a period of steady pacing at a long (≥ 500 ms) cycle length. S1 was delivered at the beginning of the simulation, and the S2 S3 interval was varied in steps of 1 ms.

### APD and CV restitution

The APD and CV restitution curves for each variant of the model were measured from a thin strip of uniform tissue 75 mm long. Four S1 stimuli were given to one end of the tissue at 500 ms intervals, followed by a premature S2 stimulus. The S1S2 interval was reduced until a propagating action potential could not be elicited. APD was measured to 90% repolarisation; so all measurements of APD in this paper correspond to APD_90_. We measured DI and APD in the centre of the strip. We also measured the time difference between the action potential upstroke at the stimulus site and the action potential upstroke in the centre of the strip, and used this information to calculate CV restitution. This method underestimates CV slightly, due to the action of the stimulus with the tissue edge.

## Results

### APD and CV restitution

**Figure 2:**
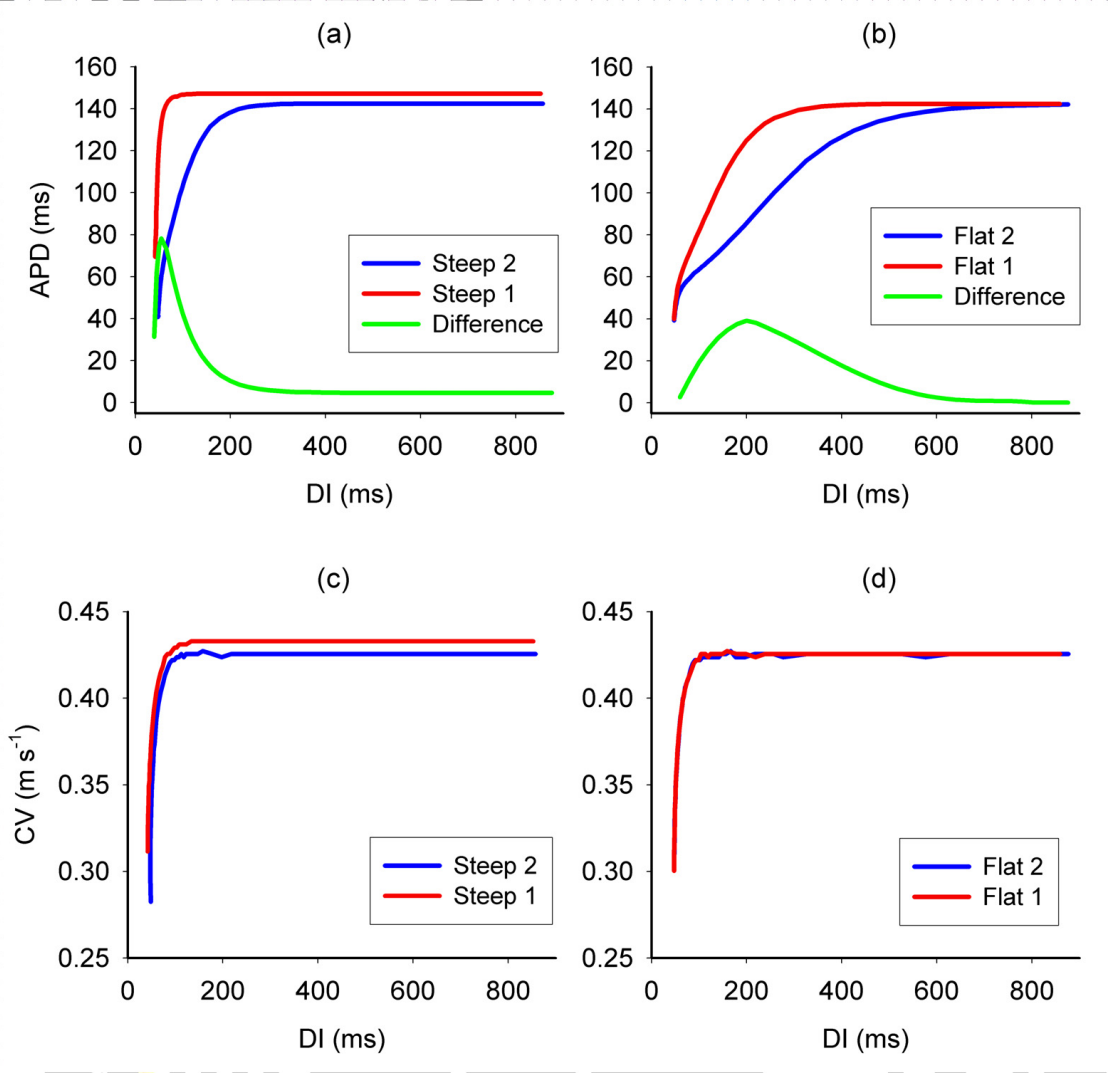
APD and CV restitution measured in a thin strip of uniform tissue. Panels (a) and (b) show APD restitution for the steep and flat variants of the model, and in each case the green line indicates the difference in APD produced at each DI. Panels (c) and (d) show the CV restitution for the steep and flat variants of the model. Model variants with restitution shown in red were allocated to region R1, and those shown in blue allocated to region R2. S3 is blocked in R1, because the tissue is still repolarising from the S2 action potential.

The APD and CV restitution for each of the four variants of the computational model measured in a thin strip of homogenous tissue are shown in Figure 2. There was little difference between the CV restitution curves. At long DI, the APD of each variant was around 140 ms, a value comparable with that found in guinea pig [24] or rabbit [16] ventricular tissue. At shorter DI, each pair of APD restitution curves diverged, showing that a markedly different APD would be elicited from each of the two variants.

### Wavebreak and re-entry

**Figure 3:**
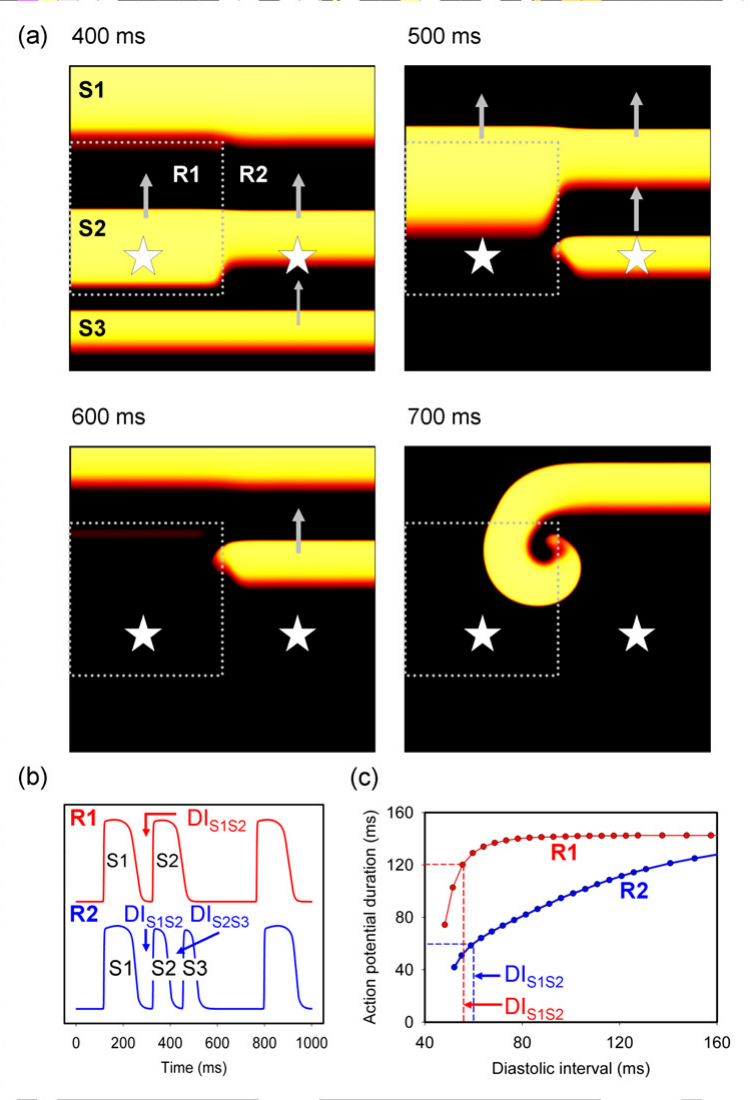
(a) Snapshots showing distribution of membrane potential in the 2D model 400, 500, 600, and 700 ms after delivery of S1 stimulus. S1 S2 interval was 200 ms, and S2 S3 interval 120 ms. The colour scheme shows resting tissue as black, and brighter colours show depolarised tissue. Dotted grey lines show boundary between R1 and R2 regions with different APD restitution, and grey arrows show direction of propagation. (b) Recordings of membrane potential from sites indicated by stars in (a). (c) APD restitution curves for R1 and R2, showing DI_S1S2_, and the APD produced in R1 and R2. See text for details.

In the tissue sheet model with heterogenous APD restitution, we were able to initiate wavebreak and re-entry over a range of S1 S2 S3 intervals when R1 and R2 had different APD restitution. This was the case when the slope of APD restitution in both R1 and R2 was steep, and also when the slope of APD restitution in both R1 and R2 was relatively flat. We were also able to initiate wavebreak and re-entry for both square and circular configurations of R1. Figure 3 shows an example of wavebreak and re-entry for the square configuration, where both R1 and R2 had steep APD restitution.

Figure 3(a) shows four snapshots of the distribution of membrane voltage within the tissue at different times after the S3 stimulus. The action potentials initiated by each stimulus propagated from the bottom to the top of the virtual tissue; hence the first panel of Figure 3(a) shows action potentials elicited by each of the S1, S2, and S3 stimuli propagating from bottom to top as indicated by the arrows. The dotted grey line indicates the boundary between regions R1 and R2. The S2 action potential has a longer APD in R1 compared to R2, and this results in block of the S3 action potential close to the bottom edge of R1 and the development of wavebreak and then a re-entrant spiral wave, shown in subsequent panels of Figure 3(a).

The two stars in each panel of Figure 3(a) indicate points from which simulated transmembrane potentials were recorded from the model, and the time series of these are shown in Figure 3(b). The top trace shows the recording from R1 and the bottom trace the recording from R2. The APD restitution of each region is shown in Figure 3(c). The first (S1) action potential elicited APDs of 139 and 142 ms in R1 and R2 respectively, corresponding to the flat regions of the restitution curves in Figure 2. The second (S2) action potential had a DI (*DI*_*S1S2*_) of 58 and 61 ms in R1 and R2, and the resulting APDs were 120 and 61 ms respectively as indicated by the dashed lines in Figure 3(c). The third (S3) stimulus was delivered 120 ms after the second, and a propagating action potential was initiated in the lower half of the virtual tissue as shown in the first panel of Figure 3(a), with a DI (*DI*_*S2S3*_) of 58 ms. Figure 3(b) shows that this action potential arrived at the R1 R2 boundary before repolarisation was complete, and so was blocked. In R2 repolarisation was complete, and so the S3 action potential continued to propagate with a wavebreak at the boundary between R1 and R2.

### Re-entry

**Figure 4:**
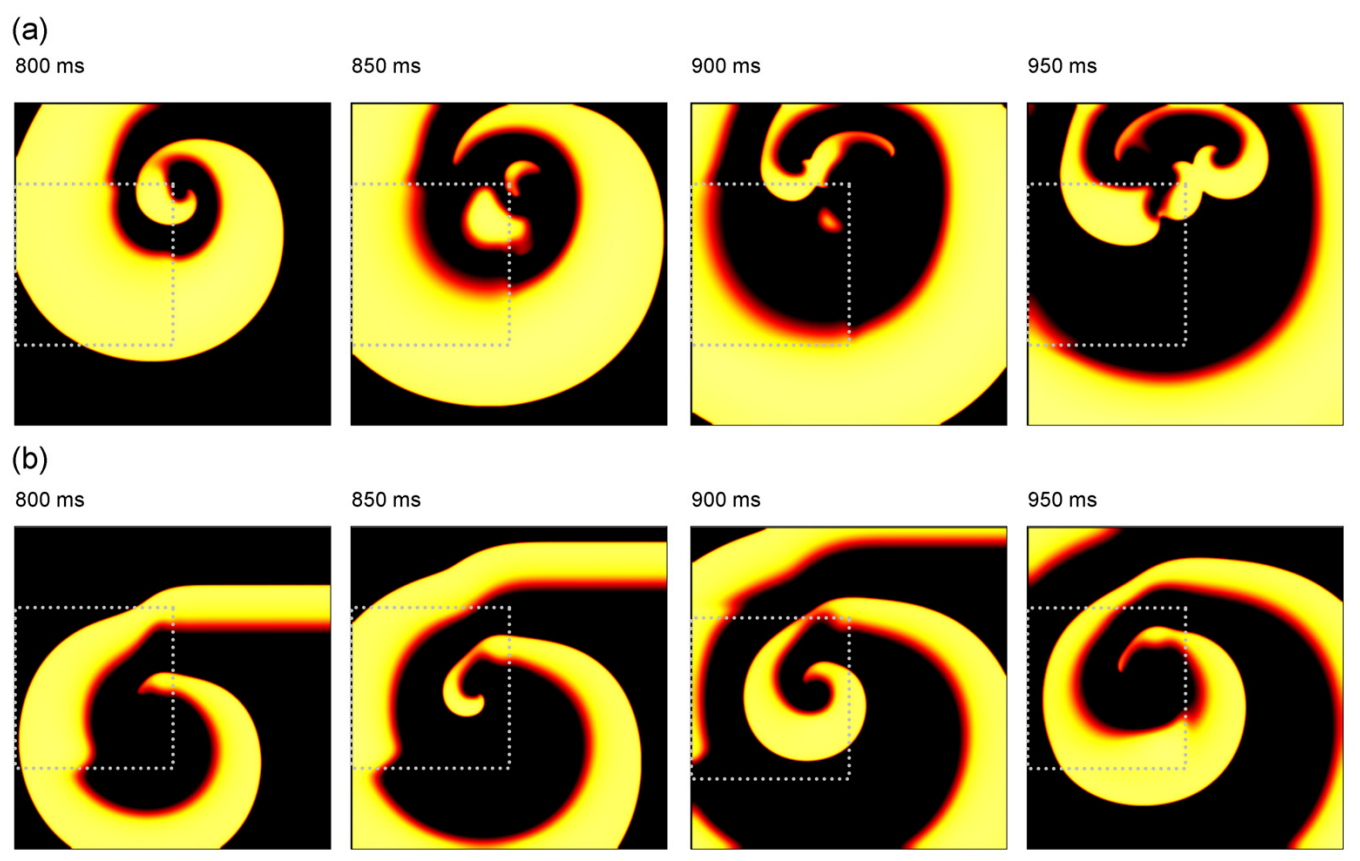
Snapshots showing the behaviour of re-entry following wavebreak. The colour scheme is the same as in Figure 3, with brighter colours showing depolarised tissue. (a) Immediate break up of re-entry for model with steep APD restitution. (b) Initially stable re-entry in model with flat APD restitution. Movies of these simulations are included in additional files 1 and 2.

In all of the simulations the wavebreak formed by block in R1 curled around the top edge of R1 and re-entered this region as it recovered, and so wavebreak always resulted in at least one cycle of re-entry. The re-entrant spiral waves tended to break up in tissue with steep APD restitution and tended to remain stable in tissue with flat APD restitution, although the simulations only extended for a few cycles of re-entry following initiation. This behaviour is illustrated in Figure 4, and also in the additional movie files.

### Size and shape of heterogeneity

The overall behaviour of virtual tissue with a circular region R1 was similar to that of virtual tissues with a rectangular region R1. For an S1 S2 interval of 200 ms, wavebreak and re-entry were initiated when the S2 S3 interval was greater than 101 ms. The upper limit of the S2 S3 interval that resulted in wavebreak was 150 ms for and R1 radius of 12.5 mm, 161 ms for radius 25 mm, and 158 ms for radius 50 mm. This upper limit compared with a value of 161 ms for the square configuration.

**Figure 5:**
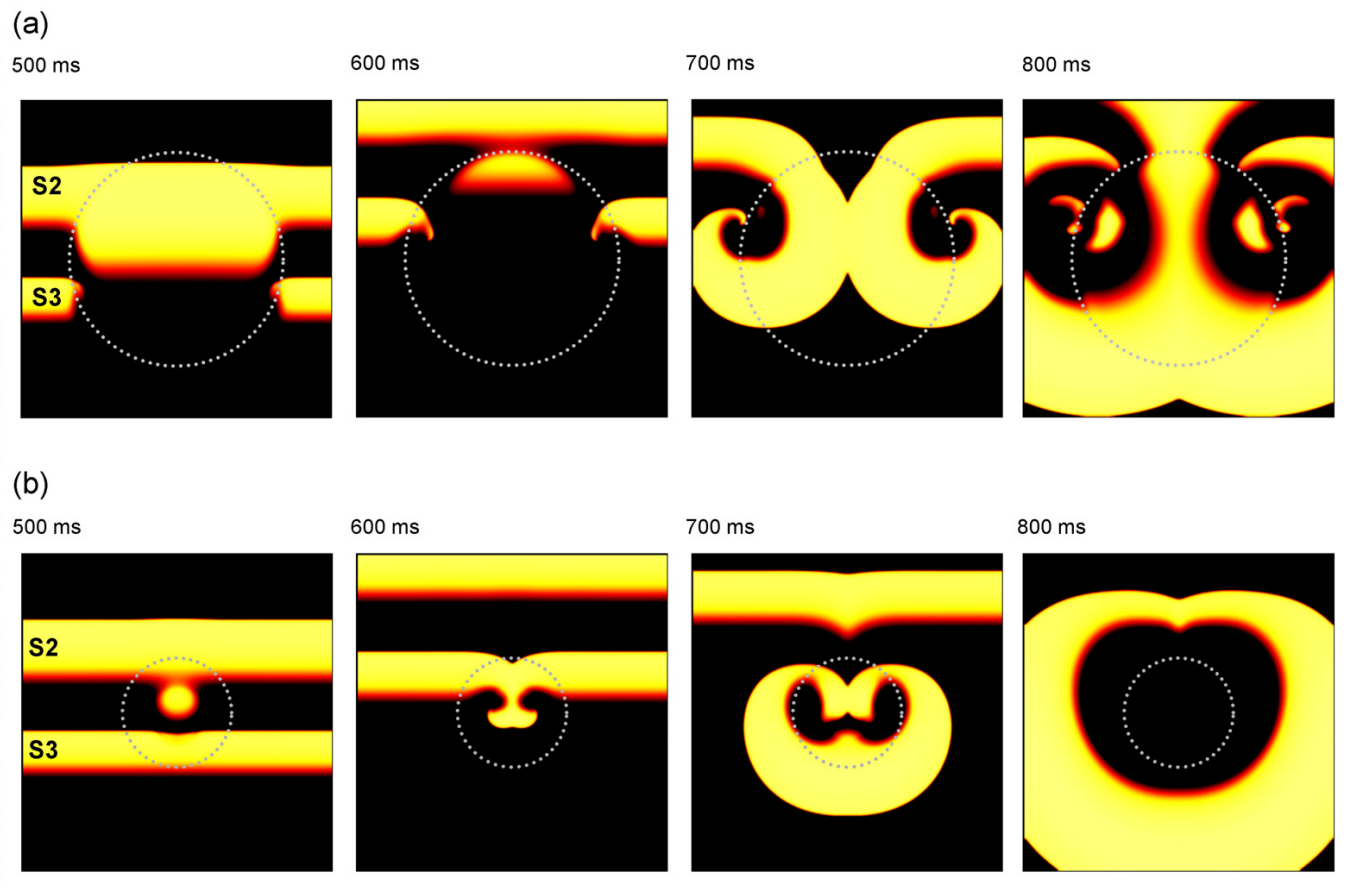
Example snapshots showing wavebreak and re-entry in simulated tissue with circular heterogeneity, and steep APD restitution. The colour scheme is the same as in Figure 3, with brighter colours showing depolarised tissue. The boundary of R1 is shown by the grey dotted line. Each panel shows consecutive snapshots of membrane voltage following stimuli with S1 S2 interval of 200 ms, and S2 S3 interval of 120 ms. (a) Wavebreak and sustained re-entry for R1 with radius of 50 mm. (b) Wavebreak and one cycle of re-entry for R1 with radius of 12.5 mm. Movies of these simulations are included in additional files 3 and 4.

An example of the initiation of re-entry for R1 radii of 12.5 and 50 mm, and an S2 S3 interval of 120 ms is shown in Figure 5 for the configuration with steep APD restitution. As in Figure 3, the boundary between R1 and R2 is indicated by a grey dotted line. In the top panel, action potentials resulting from the S2 and S3 stimuli are shown, and the prolonged action potential in R1 can be seen. For an R1 radius of 12.5 mm (Figure 5(a)), the effect of electrotonic current flow during repolarisation resulted in a smaller region with longer APD. Although this region was large enough to initiate wavebreak and 1 cycle of re-entry, the two re-entrant waves were blocked during their second cycle, and re-entry terminated. For the larger scale heterogeneity (Figure 5(b)), re-entry persisted and broke up into multiple wavelets close to the boundary between R1 and R2. Additional files 1,2,3,4show movies of the simulations shown in Figure 5.

From these findings we concluded that a circular R1 region behaves in a similar way to a square region, and that the size of the circle is small enough for electrotonic effects to become important. This latter observation is in agreement with other studies that have examined electrotonic effects with static differences in APD [10,25].

### Stimulus protocol

**Figure 6:**
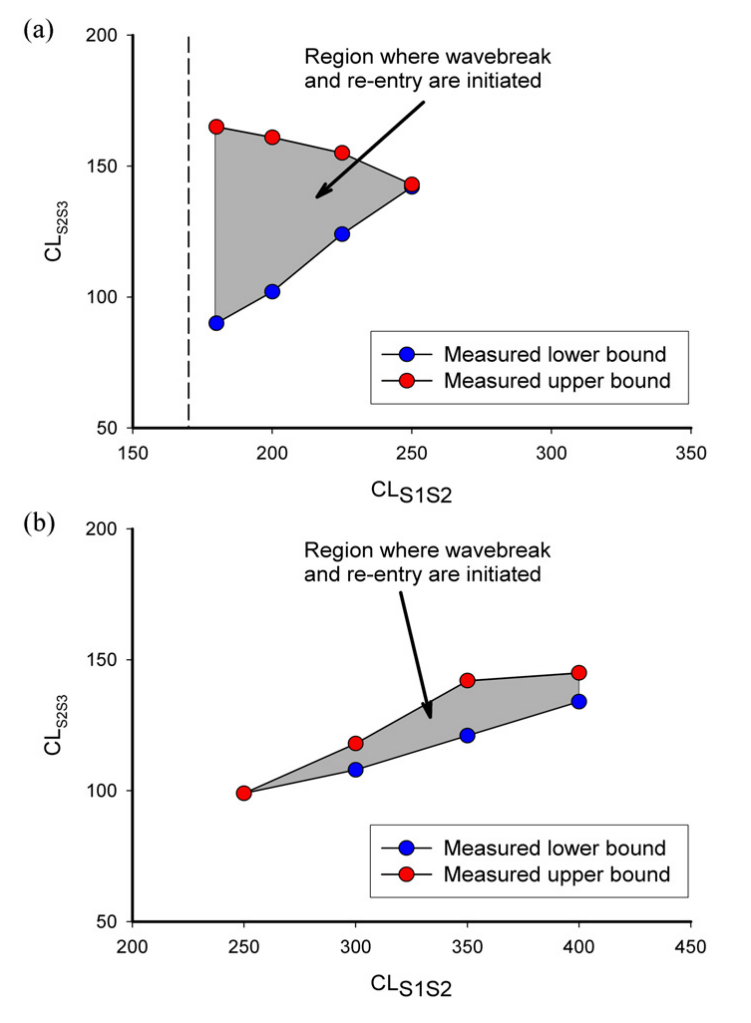
Combinations of S1 S2 interval (CL_S1S2_) and S2 S3 interval (CL_S2S3_) resulting in wavebreak and re-entry in models with (a) steep APD restitution, and (b) flat APD restitution. Red points show the upper limit of S2 S3 interval that resulted in wavebreak, above this threshold the S3 action potential propagated in region R1 without wavebreak. Blue points show the lower limit of S2 S3 interval that resulted in wavebreak, below this threshold the S3 action potential was blocked in region R2 at the stimulus site. The vulnerable regions where re-entry and wavebreak were initiated are bounded by these upper and lower limits, and are shown shaded grey. The dashed line in (a) indicates the shortest CL_S1S2 _that produced a propagating action potential.

For the square configuration of R1 and R2, and for a range of S1 S2 intervals, we measured the range of S2 S3 intervals that produced wavebreak and re-entry, and designated this the vulnerable area. Figure 6 shows the S2 S3 interval plotted against the S1 S2 interval for simulated tissue with both steep and flat APD restitution, with the vulnerable area that elicited wavebreak and re-entry shown in grey. The shape of each vulnerable area was different. For steep APD restitution, re-entry could be initiated over a range of S1 S2 intervals of 70 ms, and a range of S2 S3 intervals of up to 75 ms. In contrast, for flat APD restitution, re-entry could be initiated over a much longer range of S1 S2 intervals of 150 ms, but the range of S2 S3 intervals was much shorter, with a maximum of 21 ms. Hence, although re-entry could be initiated in tissue with both steep and flat APD restitution, the shape of the APD restitution curve was important for determining the dimensions of the vulnerable area.

A clue about these differences can be gleaned from Figure 2. Figure 2a–b indicates the difference in APD between each pair of models. For steep APD restitution, large differences in APD were produced over a narrow range of DI, whereas for flat APD restitution, small differences in APD were produced over a wider range of DI. Hence for the model with steep APD restitution, regional differences in APD could be produced only over a narrow range of S1S2 interval. In contrast, the models with shallow APD restitution produced smaller APD differences, but over a longer range of S1 S2 interval.

Figures 2 and 6 therefore highlight the key mechanism by which wavebreak and re-entry were produced by a two-stage mechanism in tissue with regional differences in APD restitution. First, a premature beat produced regional differences in repolarisation. Second, a further premature beat was partially blocked in the regions of prolonged repolarisation. The differences between the APD restitution in each region, and not their slope, determined the dimensions of the vulnerable area.

## Analysis

The broader aim of this study was to assess whether knowledge of restitution properties could be used to predict the vulnerability to re-entry shown in Figure 6. APD and CV restitution are complex properties of cardiac tissue, and the APD and CV of a particular beat are influenced by the pacing history or cardiac memory [26,27], by electrotonic current within the tissue [18], and APD may not always be a monotonic function of DI [27]. For the sake of simplicity in the analysis below, this complexity is noted but not included. In this simplified case, the APD of beat *n+1 *depends only on the preceding DI according to the iterative relation below.

*APD*_*n+1 *_= *f*(*DI*_*n*_)

*DI*_*n *_= *CL*_*n *_- *APD*_*n *_    (2)

Where *f(DI) *is the APD restitution curve that gives APD as a function of DI. For the S1 S2 S3 protocol used in this study there are two cycle lengths, *CL*_*S1S2*_, and *CL*_*S2S3 *_with corresponding diastolic intervals

*DI*_*S1S2 *_= *CL*_*S1S2 *_– *APD*_*S1*_

*DI*_*S2S3 *_= *CL*_*S2S3 *_– *f *(*CL*_*S1S2 *_– *APD*_*S1 *_)    (3)

The second diastolic interval *DI*_*S2S3 *_depends on *APD*_*S2*_, which can be calculated from the APD restitution curve. The third (S3) beat will be blocked if *DI*_*S2S3 *_is less than *DI*_*min*_, where *DI*_*min *_is the shortest DI that results in a propagating beat. If we consider tissue with regions R1 and R2 as shown in Figure 1, and ignore the effects of CV restitution, then the S3 action potential will be blocked in region R1 if



and in region R2 if



For re-entry to be initiated, we require block in region R1, and propagation in region R2. This condition produces a wavebreak, and is fulfilled if



Based on this analysis, if the APD restitution in regions R1 and R2 is identical, then re-entry cannot be initiated with this stimulus protocol. This is reflected in equation (6), where the range of *CL*_*S2S3 *_that result in re-entry is zero if both sides of the inequality are equal. Another consequence of this analysis is that wavebreak will only occur if the left hand side of equation 6 is less than the right hand side, so APD restitution must produce a longer APD in R1 than in R2 for short DI. Our final observation from this equation is that there is no requirement that the slope of any of the restitution curves should be >1, and this is supported by our findings shown in Figure 6.

**Figure 7:**
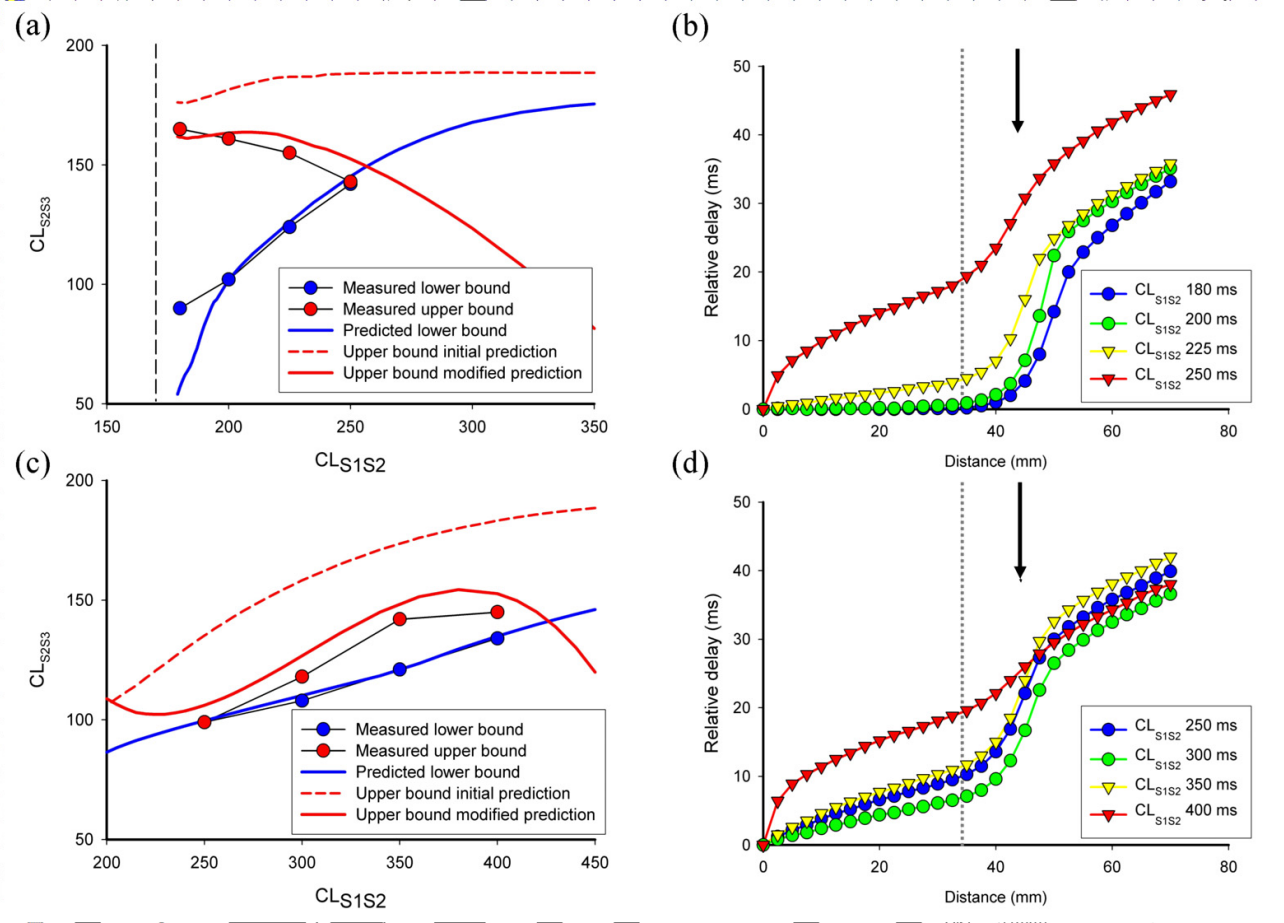
Predicted upper and lower bounds for initiating wavebreak in models. (a) Predicted upper (red) and lower (blue) bounds for model with steep APD restitution. The modified upper bound (solid red line) used information from the delays shown in (b). (b) Conduction delays measured in heterogenous tissue with two regions of steep APD restitution. Delays are delay_S2S3_, for S3 action potentials at CL_S2S3 _close to those that result in block in R1, and each line shows this delay for a different S1 S2 interval. Region R2 is to the left of the dotted grey line, and region R1 to the right. See text for details. (c) Predicted upper (red) and lower (blue) bounds for model with flat APD restitution. The modified upper bound (solid red line) used information from the delays shown in (d). (d) Conduction delays measured in heterogeneous tissue with flat restitution. See text for details.

Figure 7(a) and Figure 7(c) show the upper and lower bounds of the vulnerable region predicted from equation 6, as well as the measurements from the model, for both steep and flat APD restitution. Although the predicted lower bounds (blue lines) agreed well with the observations, the initial prediction of the upper bound (dashed red line) overestimated the range of *CL*_*S2S3 *_resulting in wavebreak, especially for longer *CL*_*S1S2*_.

Although the CV restitution of the models only exerts an effect at short DI (Figure 2), we hypothesised that conduction delays associated with CV restitution accounted for the difference between the predicted and observed upper bound, and we modified equation 4 accordingly.



The delays in this equation result from CV restitution, and depend on the distance between the stimulus site and the border of the heterogeneity. We measured these time delays for combinations of *CL*_*S1S2 *_and *CL*_*S2S3 *_that resulted in propagation of the S3 action potential, and hence corresponded to the upper bound of vulnerability. The first delay *delay*_*S1S2 *_was small except for values of *CL*_*S1S2 *_close to the lower limit, by the second delay *delay*_*S2S3*_, and these delays are plotted for steep and flat restitution in Figure 7(b) and Figure 7(d) respectively. These plots show evidence of very slow conduction in the border zone between R1 and R2. For a shorter *CL*_*S2S3*_, the S3 action potential was blocked in R2 close to the point of slowest conduction, at around 47.5 mm, indicated by a black arrow on Figures 7(b) and 7(d). We fitted a polynomial to the measurements of *delay*_*S2S3 *_at this distance to estimate *delay*_*S2S3 *_for the upper bound of vulnerability as a function of *CL*_*S1S2*_. This estimate, together with measurements of *delay*_*S1S2 *_enabled us to plot equation 7, and this is shown as a solid red line on Figures 7(a) and 7(c). This modified prediction of the upper bound provides a much better fit to the measurements. It is likely that electrotonic interaction, and errors in the polynomial fit could account for the remaining differences.

Thus our analysis of these results shows that information about both APD and CV restitution are necessary to predict the vulnerability of tissue with heterogenous APD restitution. The effect of CV restitution is to introduce delays that reduce vulnerability, and these delays depend on the distance between the stimulus site and the border of the heterogeneity. This dependence was investigated for a model of tissue with a static APD heterogeneity by Panfilov and Vasiev, who showed that the width of the vulnerable period decreases monotonically with increasing distance between stimulus site and border of the heterogeneity [9].

## Discussion

The novel finding of this study is that regional differences in APD restitution can act as a potent arrhythmogenic substrate by producing rate dependent regional differences in repolarisation. These regional differences may be concealed at normal heart rates, but exposed by a premature beat. A further premature beat can then be partially blocked by regions of refractory tissue, resulting in wavebreak and re-entry. This finding is important because the exact mechanism by which re-entry and VF are initiated in the human heart remains unclear. As a consequence, it is difficult to identify with precision those patients who are at risk of sudden cardiac death.

The arrhythmogenic effects of regional differences in repolarisation have been studied both experimentally [5,6] and with an early computational model [7]. Regions with longer refractory periods can block a premature stimulus, resulting in re-entry, and a recent computational study has examined this mechanism in detail [10]. In this study, regional differences in repolarisation were presumed to be a static feature resulting from regional pathology. Winfree [28] showed that the interaction of a premature beat with repolarising tissue was another mechanism capable of initiating re-entry, and this idea was later verified experimentally [29]. This approach was significant because it explained how re-entry could be initiated in normal, electrically uniform tissue. Another mechanism capable of producing re-entry in uniform tissue depends on APD and CV restitution. Steep restitution can produce spatial and/or temporal APD alternans leading to wavebreak and unstable re-entry [12,30].

VF and other re-entrant arrhythmias are, however, more prevalent in hearts that are affected by disease processes that augment electrical heterogeneity. For example, regional ischaemia is an effective arrhythmogenic substrate and a recent experimental study has shown that it produces regional differences in APD restitution [17]. Our present study builds on this and other experimental work [18,19].

### Mechanism

The key arrhythmogenic mechanism we have investigated is exposure of regional differences in repolarisation by a closely coupled stimulus, followed by regional block of a further closely coupled premature beat. There is no general requirement that for the slopes of the APD restitution curves to be steep, and we have shown that this mechanism produces wavebreak for both steep (slope>1) and shallow (slope < 1) APD restitution. However, the relative steepness of the APD restitution curve in each region is important, as described below.

### Importance of stimulus sites

For the sake of clarity all three stimuli were delivered from the same site in our simulations. In this scheme (Figure 1) it was important that the R1 region(s) distal to the stimulus site should have steeper APD restitution than the surrounding R2 tissue, so that the S3 beat was not blocked close to the stimulus site. Other schemes are of course possible, and in real tissue the normal and premature beats may originate from two or more different sites. This would modify the detail of the mechanism we have described, but the core idea would remain the same.

For example, if the R1 region had shallower APD restitution than the R2 region, then delivery of the S1 and S2 stimuli from within R2 would result in a long APD in R2 but a short APD in R1. Thus if the S3 beat originated in R2, it would either propagate normally with a short APD in R1, or be blocked in R2. However, if the S3 beat was to originate in R1, it could be blocked by the longer repolarisation in R2 while propagating in R1, forming a wavebreak and initiating re-entry. More simulations would clarify this, but are beyond the scope of the current study.

### Geometry

The simulations in the present study have considered only two regions, R1 and R2, but the overall mechanism is also applicable to tissue with multiple regions with regional differences in APD restitution. Our findings also indicate that the shape of the different regions has a small effect on the initiation of wavebreak and re-entry, but our results for the circular heterogeneity indicate that the size of the regions is important. In a previous computational study we have shown that both size and cell-to-cell coupling determine the potency of static arrhythmogenic heterogeneities, presumably through electrotonic current flow within the tissue [10]. Figure 5 suggests that electrotonic current flow is also important for dynamically induced heterogeneity, and smaller regions produce transient wavebreak but do not support sustained re-entry.

### Effect of CV restitution

This paper has focussed on APD restitution rather than CV restitution. The four variants of the cell membrane model used in this study possessed almost identical CV restitution (Figure 2), with significant conduction delays only becoming evident at short DI. However, the analysis given above and detailed in Figure 7 indicates that both APD and CV restitution are important in determining whether wavebreak and re-entry will occur for a given stimulus sequence. This effect is greater for longer S1S2 intervals since *APD*_*S2 *_is longer in both R1 and R2, *DI*_*S1S2 *_is shorter in R1 and R2, and hence the S3 beat is more delayed. A greater delay to the S3 beat at the boundary between R1 and R2 offers more time for the tissue in R1 to repolarise, and reduces the incidence of block. This delay therefore underlies the reduction in the vulnerable region, and explains the overestimation of the vulnerable region by equation 6.

Regional differences in CV restitution as well as APD restitution would add an additional layer of complexity to the behaviours documented here, but this detailed analysis is outside the scope of this discussion.

### Predicting vulnerability

A quantitative assessment of vulnerability resulting from regional differences in APD restitution would be a valuable clinical tool. These differences would be exposed as regional differences in repolarisation when the heart is paced at short cycle lengths. A recent computational study has shown that regional differences in repolarisation produce distinctive changes in T wave shape on the electrocardiogram [31]. We would expect that regional differences in APD restitution would produce characteristic rate-dependent changes in T wave shape.

Our preliminary efforts described above and shown in Figure 7 however, suggest that although it may be possible to identify patients at risk using this type of approach, it may be difficult to estimate the extent of vulnerability. The main reason for this is the effect of conduction delays arising from CV restitution. Although the CV restitution curve for a region of tissue may be well characterised, the delays affecting a particular premature beat depend on the spatial relationship between R1, R2, and the stimulus site(s). The effect of CV restitution delays is to prolong the DI between two closely coupled beats, and the extent of the delay depends on the distance between the stimulus site and the recording site [9]. Hence without detailed knowledge of the spatial relationship between regions with altered APD restitution and the site of origin of premature beats, it may be difficult to predict the size of the vulnerable region.

### Limitations

This study has several limitations. We used a greatly simplified computational model to represent the dynamical behaviour of tissue, which does not describe the details of current flow through ion channels, pumps, and exchangers in the cell membrane, and it does not attempt to include the effects of intracellular Ca^2+ ^storage and release. Neither does it include the effects of cardiac memory. However, the model does capture the APD and CV restitution of real cells and tissue, and it is these dynamical features that are relevant for the mechanism that was explored in this study. For the sake of simplicity and clarity, we also simulated isotropic 2D sheets with abrupt changes in APD restitution between different regions, yet real ventricular tissue is both anisotropic and 3-dimensional, and spatial changes in APD restitution are likely to be gradual. We only investigated tissues with a limited range of restitution curves. All of these limitations arose from a desire to minimise the computational demands of the study.

In this study we assumed that the APD restitution of ventricular tissue could be described by a monotonic curve where APD depends solely on the preceding DI. However, APD restitution curves recorded from human hearts may have a more complex shape [27]. In addition, there is substantial evidence to suggest that APD restitution may itself be dynamic, and depend on the stimulus history and not simply on the preceding DI [26,32].

Further work with biophysically detailed models of the cardiac cell membrane, a wider range of restitution characteristics, and models of anisotropic 3D tissue will be needed to establish fully the extent to which the findings presented here could be relevant in real cardiac tissue.

### Clinical implications

This study has some important clinical implications. In the human heart, APD restitution is flattened in ischaemia [33], and steepened by adrenergic agents [34], suggesting that inhomogeneous sympathetic innervation as a result of nerve sprouting may generate heterogenous APD restitution. Cardiac drugs can also decrease APD restitution slope [2,35]. Evidence from isolated myocytes and tissue preparations also suggest that there are transmural differences in APD restitution [36], and these could act together with the mechanisms described above. These effects could in turn be further modified by additional factors including regional stretch, hypertrophy, and regional remodelling. Detailed experimental and clinical studies are now needed to establish precisely the relative importance of these factors for the arrhythmogenic substrate.

## Conclusion

This study used a simplified computational model of cardiac tissue to test the idea that regional differences in APD restitution can be a potent substrate for initiating re-entrant arrhythmias. These regional differences can be concealed at normal heart rates. A two-stage process can produce wavebreak and re-entry. First, regional differences in repolarisation can be produced by a premature beat, and second, these regional differences can then interact with a further premature beat, resulting in wavebreak and re-entry through the well-established mechanism of conduction block. Since the determinant of wavebreak is independent of APD restitution slope, we found that re-entry could be produced in simulated tissue with both steep (slope > 1) and flat (slope < 1) APD restitution.

## Authors' contributions

RHC designed the study, wrote the simulation code, ran the simulations, and wrote the manuscript. PT conceived the study, helped interpret the results, and also contributed significantly to the manuscript.

## Appendix: Three-variable model

The 3-variable Fenton Karma model has three currents, two inward (depolarising) currents corresponding broadly to Na^+ ^and Ca^2+ ^currents, and a slow outward (repolarising) current corresponding to K^+ ^currents. The membrane voltage *V*_*m *_was scaled with the resting potential *V*_*0 *_and the Nernst potential of the fast inward current *V*_*fi *_to give a dimensionless activation variable *u *that varies between 0 and 1 where



Setting *C*_*m *_to 1 μF mm^-2^, the equations of the model were



The currents *J*_*fi*_, *J*_*si *_and *J*_*so *_had units of ms^-1 ^and were given by



Where Θ denotes the Heaviside step function and Θ*(x) *is equal to *1 *for *x ≥ 0 *and *0 *for *x *<*0*.

## Supplementary Material

Additional File 1 **Figure4a.mpg **Wavebreak and re-entry in the model with steep APD restitution. The S1S2 interval was 200 ms, and the S2S3 interval was 120 ms. Movie of simulation depicted in Figure 4a. Click here for file

Additional File 2 **Figure4b.mpg **Wavebreak and re-entry in the model with flat APD restitution. The S1S2 interval was 350 ms, and the S2S3 interval was 130 ms. Movie of simulation depicted in Figure 4b. Click here for file

Additional File 3 **Figure5a.mpg **Wavebreak and re-entry in the model with steep APD restitution, and circular R1 with radius 50 mm. The S1S2 interval was 200 ms, and the S2S3 interval was 120 ms. Movie of simulation depicted in Figure 5a. Click here for file

Additional File 4 **Figure4a.mpg **Wavebreak and re-entry in the model with steep APD restitution, and circular R1 with radius 12.5 mm. The S1S2 interval was 200 ms, and the S2S3 interval was 120 ms. Movie of simulation depicted in Figure 5b. Click here for file

## References

[B1] Karma A (1993). Spiral breakup in model equations of action potential propagation in cardiac tissue. Physical Review Letters.

[B2] Garfinkel A, Kim YH, Voroshilovsky O, Qu ZL, Kil JR, Lee MH, Karagueuzian HS, Weiss JN, Chen PS (2000). Preventing ventricular fibrillation by flattening cardiac restitution. Proc Natl Acad Sci U S A.

[B3] Xie F, Qu ZL, Yang J, Baher A, Weiss JN, Garfinkel A (2004). A simulation study of the effects of cardiac anatomy in ventricular fibrillation. Journal of Clinical Investigation.

[B4] Fenton FH, Cherry EM, Hastings HM, Evans SJ (2002). Multiple mechanisms of spiral wave breakup in a model of cardiac electrical activity. Chaos.

[B5] Han J, Moe GK (1964). Nonuniform recovery of excitability in ventricular muscle. Circulation Research.

[B6] Kuo CS, Munkata K, Reddy P, Surawicz B (1983). Characteristics and possible mechanism of ventricular arrhythmia dependent on the dispersion of action potential durations. Circulation.

[B7] Moe GK, Rheinboldt WC, Abildskov JA (1964). A computer model of atrial fibrillation. American Heart Journal.

[B8] Sampson KJ, Henriquez CS (2001). Simulation and prediction of functional block in the presence of structural and functional ionic heterogeneity. American Journal of Physiology (Heart and Circulatory Physiology).

[B9] Panfilov A, Vasiev BN (1991). Vortex initiation in a heterogeneous excitable medium. Physica D.

[B10] Clayton RH, Holden AV (2005). Dispersion of cardiac action potential duration and the initiation of re-entry: A computational study. Biomedical Engineering OnLine.

[B11] Watanabe MA, Fenton FH, Evans SJ, Hastings HM, Karma A (2001). Mechanism for discordant alternans. Journal of Cardiovascular Electrophysiology.

[B12] Qu ZL, Garfinkel A, Chen PS, Weiss JN (2000). Mechanisms of discordant alternans and induction of reentry in simulated cardiac tissue. Circulation.

[B13] Gima K, Rudy Y (2002). Ionic current basis of electrocardiographic waveforms. A model study. Circulation Research.

[B14] Antzelevitch C, Nesterenko VV, Muzikant AL (2001). Influence of transmural repolarization gradients on the electrophysiology and pharmacology of ventricular myocardium. Cellular basis for the Brugada and Long QT syndromes.. Philosophical Transactions of the Royal Society of London A.

[B15] Viswanathan PC, Shaw RM, Rudy Y (1999). Effects of I-Kr and I-Ks heterogeneity on action potential duration and its rate dependence - A simulation study. Circulation.

[B16] Banville I, Gray RA (2002). Effect of action potential duration and conduction velocity restitution on alternans and the stability of arrhythmias. Journal of Cardiovascular Electrophysiology.

[B17] Yuuki K, Hosoya Y, Kubota I, Yamaki M (2004). Dynamic and not static change in ventricular repolarisation is a substrate of ventricular ischaemia on chronic ischaemic myocardium. Cardiovascular Research.

[B18] Laurita KR, Rosenbaum DS (1996). Modulation of ventricular repolarization by a premature stimulus. Role of epicardial dispersion of repolarization kinetics demonstrated by optical mapping of the intact guinea pig heart. Circulation Research.

[B19] Laurita KR, Girouard SD, Akar FG, Rosenbaum DS (1998). Modulated dispersion explains changes in arrhythmia vulnerability during premature stimulation of the heart. Circulation.

[B20] Pak HN, Hong SJ, Hwang GS, Lee HS, Park SW, Ahn JG, Ro YM, Kim YH (2004). Spatial dispersion of action potential duration restitution kinetics is associated with induction of ventricular tachycardia/fibrillation in humans. Journal of Cardiovascular Electrophysiology.

[B21] Noble D, Rudy Y (2001). Models of cardiac ventricular action potentials: iterative interaction between experiment and simulation. Philos Trans R Soc Lond Ser A-Math Phys Eng Sci.

[B22] Fenton F, Karma A (1998). Vortex dynamics in three-dimensional continuous myocardium with fibre rotation: Filament instability and fibrillation. Chaos.

[B23] Clayton RH, Holden AV (2002). A method to quantify the dynamics and complexity of re-entry in computational models of ventricular fibrillation. Physics in Medicine and Biology.

[B24] Girouard SD, Pastore JM, Laurita KR, Gregory KW, Rosenbaum DS (1996). Optical mapping in a new guinea pig model of ventricular tachycardia reveals mechanisms for multiple wavelengths in a single reentrant circuit. Circulation.

[B25] Sampson KJ, Henriquez CS (2005). Electrotonic influences on action potential duration dispersion in small hearts: a simulation study. American Journal of Physiology (Heart and Circulatory Physiology).

[B26] Hund TJ, Rudy Y (2000). Determinants of excitability in cardiac myocytes: Mechanistic investigation of memory effect. Biophys J.

[B27] Franz MR (2003). The electrical restitution curve revisited: Steep or flat slope - which is better?. Journal of Cardiovascular Electrophysiology.

[B28] Winfree AT (1983). Sudden cardiac death: A problem in topology. Scientific American.

[B29] Frazier DW, Wolf PD, Wharton JM, Tang ASL, Smith WM, Ideker RE (1989). Stimulus induced critical point. Mechanism for electrical initiation of reentry in normal canine myocardium.. Journal of Clinical Investigation.

[B30] Pastore JM, Girouard SD, Laurita KR, Akar FG, Rosenbaum DS (1999). Mechanism linking T wave alternans to the genesis of cardiac fibrillation. Circulation.

[B31] van Huysduynen BH, Swenne CA, Draisma HHM, Antoni ML, van de Vooren H, van der Wall EE, Schalij MJ (2005). Validation of ECG indices of ventricular repolarization heterogeneity: A computer simulation study. J Cardiovasc Electrophysiol.

[B32] Cherry EM, Fenton FH (2004). Suppression of alternans and conduction blocks despite steep APD restitution: Electrotonic, memory, and conduction velocity restitution effects. American Journal of Physiology (Heart and Circulatory Physiology).

[B33] Taggart P, Sutton PMI, Boyett MR, Lab M, Swanton H (1996). Human ventricular action potential during short and long cycles. Rapid modulation by ischaemia.. Circulation.

[B34] Taggart P, Sutton PMI, Chalabi Z, Boyett MR, Simon R, Elliot D, Gill GS (2003). Effect of adrenergic stimulation on action potential duration restitution in humans. Circulation.

[B35] Riccio ML, Koller ML, Gilmour RF (1999). Electrical restitution and spatiotemporal organization during ventricular fibrillation. Circulation Research.

[B36] Yan GX, Shimuzu W, Antzelevitch C (1998). Characteristics and distribution of M cells in arterially perfused canine left ventricular wedge preparations. Circulation.

